# Increasing Evidence-Based Workplace Health Promotion Best Practices in Small and Low-Wage Companies, Mason County, Washington, 2009

**Published:** 2012-04-05

**Authors:** Sharon S. Laing, Peggy A. Hannon, Barbara Williams, Jeffrey R. Harris, Amber Talburt, Sara Kimpe

**Affiliations:** Health Promotion Research Center, Department of Health Services, University of Washington, School of Public Health; University of Washington, School of Public Health, Seattle, Washington; University of Washington, School of Public Health, Seattle, Washington; University of Washington, School of Public Health, Seattle, Washington; American Cancer Society, Seattle, Washington; American Cancer Society, Seattle, Washington

## Abstract

**Introduction:**

Modifiable health risk behaviors such as physical inactivity, unhealthy eating, and tobacco use are linked to the most common chronic diseases, and chronic diseases contribute to 70% of deaths in the United States. Health risk behaviors can be reduced by helping small workplaces implement evidence-based workplace health promotion programs. The American Cancer Society's HealthLinks is a workplace health promotion program that targets 3 modifiable health risk behaviors: physical inactivity, unhealthy eating, and tobacco use. We evaluated employers' implementation of HealthLinks in small workplaces.

**Methods:**

We targeted Mason County, Washington, a rural low-income community with elevated obesity and smoking rates. We conducted baseline assessments of workplaces' implementation of program, policy, and communication best practices targeting the health risk behaviors. We offered tailored recommendations of best practices to improve priority health behaviors and helped workplaces implement HealthLinks. At 6 months postintervention, we assessed changes in best practices implementation and employers' attitude about HealthLinks.

**Results:**

Twenty-three workplaces participated in the program. From baseline to follow-up, we observed significant increases in the implementation of physical activity programs (29% to 51%, *P* = .02), health behavior policy (40% to 46%, *P* = .047), and health information communication (40% to 81%, *P* = .001). Employers favorably rated HealthLinks' appeal, relevance, and future utility.

**Conclusion:**

When offered resources and support, small and low-wage workplaces increased implementation of evidence-based workplace health promotion best practices designed to reduce modifiable health risk behaviors associated with chronic diseases. Results also suggest that HealthLinks might be a sustainable program for small workplaces with limited resources.

## Introduction

More than half of Americans have 1 or more chronic diseases such as heart disease, cancer, stroke, hypertension, and diabetes (1), and 39% of the working-age population have at least 1 chronic disease (2). Health risk behaviors such as tobacco use, unhealthy eating, and physical inactivity are linked to the most common chronic diseases, and chronic health conditions contribute to 70% of all deaths in the United States (3).

Employers face mounting health care and productivity costs from chronic illnesses among workers (4). Lost work days and lower worker productivity linked to the most common chronic health conditions may result in an annual economic loss in Washington State of $28.93 billion in 2011 (5). Low-income workers are concentrated in small workplaces, and they report more chronic diseases and higher smoking levels than other workers (6). Small workplaces are less likely than larger workplaces to provide workplace health promotion (WHP) programs (7), yet employers in small workplaces might be able to reduce modifiable health risk behaviors among their workers by implementing evidence-based WHP best practices that target health-related policies, programs, and communication (8).

The University of Washington Health Promotion Research Center (HPRC) and the American Cancer Society — Great West Division (ACS-GWD) collaborate to promote evidence-based chronic disease prevention to employers (9,10). Our research has shown that employers and human resources staff are motivated to implement evidence-based WHP programs and should be targeted in these efforts (11). ACS-GWD offers a WHP program that delivers free health promotion services to reduce modifiable health risk behaviors. The program, HealthLinks, is tailored for small workplaces with limited resources in that it provides in-person assistance from an ACS-GWD staff person who implements and supports the program through resources offered to the workplaces. This study's objectives were to 1) improve small workplaces' capacity to participate in HealthLinks, 2) increase employers' implementation of evidence-based WHP best practices in small and low-wage workplaces, and 3) evaluate employers' attitudes about WHP after participating in HealthLinks.

## Methods

### Study design

We conducted a preassessment and postassessment (no comparison group) of employers' implementation of evidence-based WHP best practices and their attitudes toward WHP after they received the HealthLinks intervention. We conducted the study from January 2009 through September 2009 in Washington State. The University of Washington Institutional Review Board exempted the study from further review after receiving a summary of the procedures and a copy of measures.

### Sample

We targeted Mason County, a largely rural community that has elevated health risk behaviors. The county reports a 29% obesity rate (2% higher than the state) and 29% current smoking rate (14% higher than the state) (12). The average income of residents in the county is 20% less than state levels, $45,417 versus $56,317 (13).

We recruited 23 small workplaces (defined as a workplace with fewer than 250 workers) in Mason County, Washington. We identified workplaces that met the inclusion criteria by using several approaches: 1) accessing a public database of businesses in the region (14); 2) obtaining referrals from the Washington State Department of Health; and 3) identifying workplaces that had a prior relationship with ACS-GWD through participation in fundraising events or other activities.

### Program procedures

HealthLinks consists of 5 steps: 1) recruitment of workplaces, 2) assessment of baseline implementation of best practices, 3) recommendation of best practices, 4) implementation of recommended best practices, and 5) assessment of employer's implementation of best practices at 6-months postintervention and their attitude about WHP. To recruit workplaces (step 1), an ACS-GWD interventionist telephoned the upper-level manager at each workplace and briefed the manager on HealthLinks. If the manager showed interest in participating in HealthLinks, the interventionist described the program in more depth. Information offered included 1) an outline of the relationships among missed work days, work productivity, and lost revenue, and 2) an outline of the relationship between WHP and return on investment. To assess baseline implementation of best practices (step 2), an hour-long, in-person assessment was conducted with the workplace manager. The ACS interventionist determined which among the WHP best practices (policy, program, and communication) were present or absent at the workplace (the best-practice instrument is in Appendix A). Two weeks after the assessment, the interventionist prepared a tailored recommendation report (step 3) that listed each best practice and noted whether the practice was fully or partially implemented. The interventionist then recommended actions to fully implement the best practices (the list of resources and services that could be recommended are in Appendix B). The ACS interventionist presented the recommendation report to the manager, who selected 2 to 4 recommended actions to implement. Working with the manager, the interventionist implemented the selected recommendations (step 4). For example, employers interested in a physical activity program might select the 10-week Active for Life (AFL) program (15,16). The interventionist and personnel from the Mason County Department of Health offered resources and training sessions for the workplace contact responsible for overseeing the AFL program. They also assisted in enrolling participants and tracking physical activity goals by using an electronic tracking system. Participants received incentives when they achieved their physical activity goals and completed AFL. Incentives included boxed lunches and gift cards to local grocery stores (eg, Top Foods, Trader Joe's, Subway). Six months after the recommendation report, HPRC staff re-administered the assessment instrument to evaluate changes in best-practice implementation from baseline to follow-up. We also assessed employers' attitudes about WHP.

### Outcome measures

We assessed employers' implementation of evidence-based best practices before and 6 months after HealthLinks by using the Employer Practices Survey, a 50-item instrument consisting of closed-ended, nonscaled questions. The survey included 12 questions on tobacco use, healthy eating, and physical activity policies; 7 items on physical activity and tobacco use cessation programs; and 19 items on communication of health information. Primary outcomes included percentage of implementation of policy, program, and communication best practices and overall best practices.

We assessed employers' perception of and satisfaction with HealthLinks at 6 months after the program by using an 8-item Employer Attitude and Satisfaction Survey comprising open- and closed-ended questions. The outcomes were 1) perceived barriers, 2) HealthLinks components employers liked most, 3) HealthLinks components most likely to affect future wellness activities, and 4) HealthLinks communication materials that were most helpful.

### Statistical analysis

We scored most of the Employer Practices Survey questions dichotomously, using a score of 1 for the practices in place and a score of 0 for those not in place. We evaluated employers' implementation of tobacco policy by using 3 values, 0, 0.75, and 1. We assigned a score of 0 if the employer had no tobacco policy. We assigned a score of 1 if the employer had a complete tobacco ban policy (eg, tobacco use was not allowed anywhere on workplace grounds or in vehicles). We assigned a score of 0.75 if the employer did not allow using tobacco in the building(s). We assigned a score of 0.75 (rather than 0.50) because, by forbidding smoking indoors, employers were restricting most workers' tobacco use for most of their working hours. For each best practice, we created a summary score by summing the values, dividing by the number of possible points, and reporting the result as a percent. We calculated an overall best-practice score for each employer by summing each best practice score and taking the mean.

We used Wilcoxon matched pairs tests to analyze significant differences in best-practice implementation from baseline to follow-up. We analyzed the data by using SPSS 14.0 for Windows (SPSS, Inc, Chicago, Illinois), and we calculated all reported significant differences at the 95% confidence level.

To assess employers' attitudes and perceptions, we calculated frequency counts for employers' responses to closed-ended questions on the Employer Attitude and Satisfaction Survey. For responses to open-ended questions, we looked for responses with similar themes and reported the most common themes.

## Results

### Workplace characteristics

We contacted 69 eligible workplaces in Mason County, Washington, and intervened with 23 (33% participation rate); the workplaces had an average of 42 workers. Most (n = 20) workplaces had 200 workers or fewer. The top 5 industries were tribal centers, lumber and forestry, financial institutions, academic institutions, and public service agencies.

### Objective 1: Improve small workplaces' capacity to participate in HealthLinks

Several factors affected workplaces' capacity to participate in HealthLinks. More than half (n = 14) of participating workplaces had a previous relationship with ACS. Two factors most likely to influence workplaces' decision to participate in HealthLinks were upper management support (n = 8) and concern about the health needs of workers (n = 7). The HealthLinks characteristics that drove employers' participation included the reputation of ACS (n = 8) and the fact that HealthLinks was easy to implement, broad in scope, and free (n = 8).

workplaces' capacity to participate in HealthLinks depended on resources received. Our intervention tracking system documented which resources and educational presentations we delivered to the workplaces (Table 1). The 3 resources that we delivered to more than half of workplaces were access to fightcancer.org, e-newsletters, and the Quit Line promotional posters. In addition, 10 workplaces and 173 workers from these sites participated in AFL; the Lunch and Learn topic that workplaces most frequently requested was physical activity (n = 9), followed by healthy eating and stress management.

### Objective 2: Increase implementation of the HealthLinks program

Overall, implementation of best practices increased significantly for all 3 practice types — policy, program, and communication (Table 2). On average, workplaces implemented 36% of the best practices at baseline and 59% at follow-up (*P* < .001).

### Objective 3: Evaluate employers' attitude about HealthLinks

At follow-up, 21 employers reported high satisfaction with HealthLinks (Table 3). The most popular HealthLinks components were the e-newsletter and the Lunch and Learn presentations. The most helpful communication material was the e-newsletter. Employers favorably rated the Lunch and Learn topics for ease of promotion, relevance, and appeal. Employers rated sessions on physical activity, healthy eating, and stress management as easiest to promote; ratings for relevance and appeal were similar (data not shown). The HealthLinks component most likely to influence future WHP decisions was the Employer Practices Survey assessment of best practices.

Of the 23 participating workplaces, 12 identified at least 1 barrier to HealthLinks implementation. The most common barrier was workers' not having the time to participate (n = 7) (Table 3).

## Discussion

In our study, we met the 3 proposed objectives: 1) improved the capacity of small workplaces to participate in the HealthLinks program, 2) implemented HealthLinks with on-site support from a respected community partner, and 3) evaluated attitudes about HealthLinks program components. Guidelines to aid employers in adopting WHP programs are available (17); other researchers have identified characteristics that make WHP programs sustainable (18). The HealthLinks program for small workplaces is potentially sustainable over time.

Sustainable WHP programs target high-risk populations, involve upper management buy-in, increase program accessibility, offer incentives, and increase health awareness through effective communication (18). HealthLinks exhibited these key elements of sustainable WHP programs for small workplaces.

We effectively targeted high-risk populations (a community with elevated rates of obesity and tobacco use). Most workplaces selected the Quit Line promotional posters, and almost half participated in the intensive physical activity program, thus showing the importance of tobacco use and weight management to the targeted workplaces. In addition, employers appeared to support HealthLinks; they rated the HealthLinks resources and services as useful, relevant, and appealing.

HealthLinks increased workers' access to AFL, with almost half of workplaces participating in the program. The high level of participation is likely attributable to the support provided by the ACS-GWD interventionist and Mason County Department of Health personnel who helped to identify incentives, managed competitive teams, and coordinated the program at workplaces. Our results demonstrate the importance of offering small workplaces hands-on support to improve workers' participation in health promotion programs, thus increasing employers' capacity to engage their workers. Without support and a champion to help promote AFL, many small workplaces may not have had the capacity to implement AFL*.*

HealthLinks also helped employers promote the free Quit Line through on-site postings, thus enhancing access to a tobacco use cessation program. Research has shown that although most large and small workplaces rank smoking cession as a priority, only 2% offer cessation benefits (19) and less than 10% of small workplaces offer cessation programming (7). Like other researchers (20), we found that workplaces did not offer tobacco use cessation benefits; however, after HealthLinks, approximately two-thirds of employers promoted the state Quit Line through posters and other print materials, and 26% received information about instituting tobacco ban policies. These are encouraging results for small workplaces. The results demonstrate the willingness of employers in small workplaces to address cessation through policy and programs when they are offered resources.

Improving workers' health education through effective communication is a key element of sustainable WHP programs (21,22) and enhances the sustainability of these programs. In our study, employers showed a high likelihood of implementing various communication strategies to improve workers' health awareness. Improved health communication was most likely due to the availability of ready-to-use materials and regular distribution of a health-based e-newsletter, making it easier for employers to offer up-to-date health information. With ACS's assistance, we helped employers establish a communication system that used diverse distribution channels (posters, e-newsletters, fightcancer.org website, Lunch and Learn health education sessions) and offered health information covering multiple topics, with the intention that the workplaces would be able to sustain health awareness among their workers after the intervention ended.

This study has several strengths. The first is that we intervened in a community with elevated smoking and obesity rates. Second, we collected both process and outcome-level data, with process-level data corroborating outcome-level results. Finally, we collaborated with a known and respected community partner, ACS, which set in motion a community-based partnership that strengthened the recruitment and intervention-delivery processes and helped to sustain the relationships with the workplaces.

The study also has several limitations. We did not collect worker-level data, and this limits our understanding of how the HealthLinks program affected workers' health behaviors and attitude. Second, our study used a preintervention and postintervention analysis without using a comparison group; however, our results demonstrated the feasibility of implementing WHP programs in small and low-wage workplaces and may potentially pave the way for future randomized controlled trials using the model of working with community partners and offering enhanced support.

Employers in small and low-wage workplaces can improve their workers' health through evidence-based WHP best practices targeting specific modifiable health risk behaviors. The keys to working with small workplaces include making the WHP program easy to implement, collaborating with a respected community partner, and offering free resources and hands-on support. By targeting high-risk communities, obtaining employer buy-in, making the health programs accessible, and effectively communicating information to workers about health and wellness, WHP programs such as HealthLinks have the potential to be sustained over time. A recent report emphasized the need to disseminate "real-life" successful, WHP programs (21). Our study showcased a WHP program tailored to small and low-wage workplaces that increased employers' implementation of evidence-based best practices. Furthermore, we targeted and reached small workplaces with workers at high risk for obesity and tobacco use.

## Figures and Tables

**Table 1.** Resources and Programs Delivered to Participating Workplaces (n = 23), Mason County, Washington, 2009^a^

**Resources and Programs**	No. of Events	No. of Workplaces
**Programs/resources received by workplaces**		
Access to American Cancer Society website: fightcancer.org	21	21
Monthly e-newsletter: *Healthy Living*	20	20
Tobacco use cessation promotion: Washington State Tobacco Quit Line posters	15	15
Educational material for healthy eating at group meetings: *Meeting Well*	11	11
Physical activity program: Active for Life	10	10
Tobacco policy implementation CD	6	6
"No smoking" signs	4	4
**Lunch and Learn presentations**		
Physical activity^b^	21	9
Healthy eating^c^	11	8
Stress management	8	7
Tobacco use cessation	4	4

a A detailed outline of the resources and services that HealthLinks offers is in Appendix B.

b Physical activity presentations were delivered 2 or more times at 7 workplaces.

c Healthy eating presentations were delivered 2 or more times at 3 workplaces.

**Table 2.** Workplaces' Implementation of Evidence-Based Best Practices (Communication, Policy, and Program) at Baseline and 6 Months Follow-Up (n = 23), Mason County, Washington, 2009^a^

**Best Practice**	Baseline, Mean % (SD)	6 Months Follow-up, Mean % (SD)	Wilcoxon Matched Pairs, *P* b
Healthy eating policy	16 (28)	24 (34)	.07
Physical activity policy	28 (25)	35 (32)	.32
Tobacco use cessation policy	76 (22)	78 (20)	.56
Policy total	40 (16)	46 (18)	.047
Program total^c^	29 (45)	51 (51)	.02
Communication total^d^	40 (28)	81 (25)	.001
Total best practices implementation	36 (23)	59 (22)	.001

a For each best practice, we created a summary score by summing the items measuring the degree of best practice implementation and dividing by the number of items; therefore, we scored each best practice as being implemented from 0% to 100%. We calculated an overall best-practice score for each workplace by summing the individual best-practice scores and taking the means.

b Although the Wilcoxon test is used for ranked scores, it was appropriate to evaluate mean values in this instance.

c We assigned scores ranging from 0 to 1 to calculate the program best-practice score. We assessed only tobacco use cessation and physical activity for the program best practice; no company reported tobacco use cessation programming and, therefore, we show physical activity program results only. Physical activity program includes Active for Life and other physical activity programs.

d Communication total consists of frequency of health topic communicated, number of topics communicated, number of channels used to communicate the health topic (eg, electronic information, printed materials, formal presentations at workplaces), and the promotion of the Washington State Tobacco Quit Line program.

**Table 3.** Employers' View and Perception of Workplace Health Promotion at 6 Months After the HealthLinks Intervention (n = 23), Mason County, Washington, 2009^a^

**Category**	n
**Employers' views**	
Employers encountered barriers to choosing a health program^b^	12
Workers unable to participate due to lack of time	7
Program offered is not relevant (nonsmokers at the workplace)	1
Difficult to engage smoking workers	1
Company undergoing changes	1
Difficult to navigate Active For Life website	1
No specific reason offered	1
Employers did not encounter barriers to choosing a health program	11
**Employers' perception**	
Component that employers liked the most^c^	
E-newsletter	8
Lunch and Learn presentations	7
Assessment	3
Recommendation report	1
Active for Life^d^	1
All components	1
Posters	0
No response provided	2
**Component that employers perceived as most likely to affect thinking and planning**	
Assessment	7
Lunch and learn presentations	4
E-newsletter	4
Recommendation report	3
All components	2
Active for Life	0
Posters	0
No response provided	3
Materials that were most helpful	
E-newsletter	14
Fightcancer.org	3
Washington State Tobacco Quit Line referral	1
* Meeting Well* promotional material	0
No response provided	5

a For each item presented in this table, choices were presented to the respondent (as indicated) unless otherwise specified.

b Open-ended question.

c Respondents selected multiple choices for this item.

d Less than one-half of worksites (10 of 23) participated in physical activity programming.

## Appendices

### Appendix A. American Cancer Society HealthLinks Washington Employer Practices Survey

This appendix is available for download as a Microsoft Word file [DOC - 126 K]

### Appendix B. Description of Resources and Educational Presentations

Resources and services that were recommended to employers to support each of the best practices that HealthLinks promotes are listed below.

#### Policy

*Meeting Well*: An information packet about planning a healthy menu for staff events.

Tobacco policy CD: An information CD to help workplaces develop and implement partial or complete tobacco use ban policies.

"No smoking" signs: Signs that workplaces can post to discourage tobacco use and reinforce written tobacco use cessation policies.

#### Programs

Active for Life physical activity program: A 10-week, workplace, group-based physical activity program. The intervention included goal setting, selfmonitoring, incentives, and team competition. An interventionist from the American Cancer Society (ACS) — Great West Division and personnel from the Mason County Department of Health worked with each workplace to implement and coordinate the program.

#### Communication

*Healthy Living* e-newsletter: A monthly ACS-produced publication with health tips that employers can distribute electronically or as hard copy.

Fightcancer.org: An ACS-operated website that offers comprehensive information to workers for maintaining a healthy lifestyle.

Lunch and Learn presentations: ACS interventionist-administered, 30-minute lunchtime presentations with practical information on healthy eating, physical activity, tobacco use cessation, and stress management.

Washington State Tobacco Quit Line: The Quit Line is a state-funded tobacco use cessation service with expert counseling and nicotine replacement therapy for uninsured, low-income people. The Quit Line was promoted at the workplaces by using large posters, a referral form for workers interested in joining the Quit Line, and Lunch and Learn presentations. Workers who contact the Quit Line may also receive information about additional state-run cessation resources that were not part of the HealthLinks program.
