# Supplementary material for: Increasing Evidence-Based Workplace Health Promotion Best Practices in Small and Low-Wage Companies, Mason County, Washington, 2009
Source: Prev Chronic Dis. 2012 Apr 5;9:E83. (PMC3396550)
Supplement: Supplementary file 1 [file 11_0186_01.doc]

**Appendix A**

**
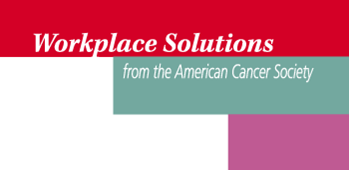
**

American Cancer Society

***HealthLinks Washington***

Employer Practices Survey

Organization:

Respondent(s):

Date: _______

The survey we’re doing today addresses prevention of cancer and other chronic diseases at the workplace. The questions correspond to best practices that affect key health behaviors like tobacco use, physical activity, nutrition, and cancer screening. There are five sections in the survey: Health Insurance Benefits, Workplace Policies, Employee Programs, Tracking, and Communication.

**Health Insurance Benefits**

This first section is about this organization’s **health insurance benefits**.

What is the name of the health plan that **most** of the employees subscribe to? _______

For these questions, please answer about the **current** benefits with this health plan.

Let’s begin. For this first set of questions, please answer **yes or no**.

| **Question** | | **Yes** | **No** |
| --- | --- | --- | --- |
| **1** | Does this organization pay for at least 50% of the total cost of personal health insurance for all full time employees? | 1 | 0 |
| **2** | Does this organization pay for at least 50% of the total cost for family coverage of all full time employees? | 1 | 0 |

Do you have control over the benefit design for that plan? Yes____ No_____

*If yes, proceed to next question on the survey*.

*If no, proceed to ‘Workplace Policies’ section of the survey on page 2.*

For the next set of questions, there will be **three answer choices**. For each preventive care service I list, please tell me whether it is **covered in this organization’s primary health plan with** **no out-of-pocket expense, or covered with an out-of-pocket expense, or not covered at all**. By **out-of-pocket expense**, I mean a co-pay, co-insurance, or deductible the employee is required to pay.

| **Preventive Service** | | **Covered with no out of pocket expense** | **Covered with an out-of-pocket expense** | **Not Covered** |
| --- | --- | --- | --- | --- |
| **3a** | Breast cancer screening, or mammogram | 1 | 2 | 3 |
| **3b** | Cervical cancer screening, or Pap smear test | 1 | 2 | 3 |
| **3c** | Colon cancer screening, such as colonoscopy or the fecal occult blood test | 1 | 2 | 3 |
| **3d** | Prescription smoking cessation medication, such as Zyban®, Wellbutrin®, and Chantix® | 1 | 2 | 3 |
| **3e** | Over-the-counter nicotine replacement therapy, including nicotine patch, lozenges, and gum | 1 | 2 | 3 |
| **3f** | Face-to-face tobacco cessation counseling, including group and individual counseling | 1 | 2 | 3 |

**Workplace Policies**

The second section is about the organization’s workplace policies. The questions are about tobacco, physical activity, nutrition, and sun protection.

For most of the questions in this section, please answer **yes or no**.

For these questions, it’s important that we discuss only policies that affect most of this organization’s employees. **Please answer “yes” to a question only if it applies to at least 75% of the organization’s employees. If a policy affects less than 75% of the employees, please answer “no”.**

**First, I will read two statements describing tobacco use policies. Please tell me which one most closely describes this organization’s current policy.**

| **4** | Employees are allowed to use tobacco on the premises, including in vehicles, but not inside the building. | 1 |
| --- | --- | --- |
|  | Employees and visitors are banned from using any form of tobacco throughout the premises, including company grounds and vehicles. | 2 |

| **Question** | | **Yes** | **No** |
| --- | --- | --- | --- |
| **5** | Does this organization have any written policy restricting employee tobacco use? | 1 | 0 |
| **If yes, go to #6. If no, go to #7.** | | | |
| **6** | Does this organization have enforcement procedures for its policy on tobacco use? | 1 | 0 |
| **7** | Does this organization provide food on-site, such as cafeterias or vending machines? | 1 | 0 |
| **If yes, go to #8. If no, go to #10.** | | | |
| **8** | Are healthy food choices available on-site, such as fruit, vegetables, or low-calorie foods? | 1 | 0 |
| **If yes, go to #9. If no, go to #10.** | | | |
| **9** | Are healthy food choices subsidized or priced competitively? | 1 | 0 |
| **10** | Does this organization label healthy food choices, or post any nutritional content about healthy food choices? | 1 | 0 |
| **11** | Is there an organizational policy stating healthy foods will be provided at meetings and other employee events? | 1 | 0 |
| **12** | Does this organization provide access to physical activity facilities, such as walking trails or fitness areas, on-site or nearby? | 1 | 0 |
| **13** | Has this organization posted "take the stairs" signs near elevators and stairwells? Not Applicable | 1 | 0 |
| **14** | Has this organization negotiated discounts or financial incentives for employees to join commercial fitness centers? | 1 | 0 |
| **15** | Do any of this organization’s employees work primarily outdoors? | 1 | 0 |
| **If yes, go to #16. If no, go to #18.** | | | |
| **16** | Does this organization have a policy requiring employees to use sun protection, such as sunscreen or protective clothing, if they are exposed to sun while on the job? | 1 | 0 |
| **17** | Does this organization provide sunscreen or protective clothing for outdoor workers? | 1 | 0 |

**Employee Programs**

This third section is about this organization’s **health programs.** These are programs that the organization offers, or contracts with a vendor to offer to employees. These are separate from insurance benefits. **The questions will be about programs for tobacco cessation and physical activity. Again, please answer “yes” only if at least 75% of employees have access to the program.**

| **Question** | | **Yes** | **No** |
| --- | --- | --- | --- |
| **18** | Does your organization contract with an outside vendor to provide an employee telephone tobacco cessation counseling program, or quitline? | 1 | 0 |
| **If yes, go to #19. If no, go to #21.** | | | |
| **19** | Does the telephone counseling service provide nicotine replacement therapy? | 1 | 0 |
| **If yes, go to #20. If no, go to #21.** | | | |
| **20** | Is there any out-of-pocket expense for the nicotine replacement therapy? | 1 | 0 |
|  |  |  |  |
| **21** | In the past year, has your company organized or sponsored an employee physical activity program? | 1 | 0 |
| **If yes, go to #22. If no, go to #25.** | | | |
| **22** | Does the program allow participants to set their own physical activity goals? | 1 | 0 |
| **23** | Is the physical activity program group-based? | 1 | 0 |
| **24** | Does the program offer incentives to participate? | 1 | 0 |

**Tracking**

**The fourth section is about employee health surveys and other activities that your organization may conduct in order to measure and track health-related issues among your employees**.

| **Question** | | **Yes** | **No** |
| --- | --- | --- | --- |
| **25** | Has your organization conducted an employee health risk appraisal or surveyed employees about health risk behaviors, or contracted with a vendor to do this? | 1 | 0 |
| **If yes, go to #26. If no, go to #27.** | | | |
| **26** | About what percentage of all employees completed the survey? | _____% | |

**Communication**

**The last section is about health promotion and communication. By this I mean your organization’s efforts to communicate with employees specifically about health, such as health status, health behaviors, or chronic disease prevention. This could be anything from a newsletter or email to an organized campaign or group event. These are communications outside of health insurance open enrollment periods or insurance provider communications**.

| **Question** | | **Yes** | **No** |
| --- | --- | --- | --- |
|  |  |  |  |
| **27** | Does your organization communicate with employees about health at least four times a year? | 1 | 0 |
| **28** | Does your organization make any health-related materials regularly available to employees, such as newsletters, pamphlets, or websites? | 1 | 0 |
| **29** | Does your organization provide health topic informational sessions, such as lunch-and-learn presentations, to your employees? | 1 | 0 |
| **30** | Does your organization promote the WA state-sponsored tobacco cessation quitline among employees? | 1 | 0 |
| **31** | Does your organization promote the WA-state breast and cervical cancer program among employees? | 1 | 0 |

Now, I will list some **methods for communicating with employees**. For each one, please tell me if you use this method to communicate with employees about health issues.

| **Item** | | **Yes** | **No** | | |  |
| --- | --- | --- | --- | --- | --- | --- |
| **32a** | Website | 1 | 0 | | |  |
| **32b** | Email | 1 | 0 | | |  |
| **32c** | Newsletters | 1 | 0 | | |  |
| **32d** | Bulletin boards | 1 | 0 | | |  |
| **32e** | Payroll stuffers | 1 | 0 | | |  |
| **32f** | Mailings to employees’ homes | 1 | 0 | | |  |
| **32g** | Meetings | 1 | 0 | | |  |
| **32h** | Other(s): |  |  | | |  |
|  |  | | | | |  |
| Now I will list some **health behaviors.**  For each one, please answer “yes” if this organization has **communicated** with employees about the health behavior in the past year. | | | | | | |
| **Item** | | | | **Yes** | **No** | |
| **33a** | Tobacco cessation | | | 1 | 0 | |
| **33b** | Nutrition | | | 1 | 0 | |
| **33c** | Physical activity | | | 1 | 0 | |
| **33d** | Cancer screening | | | 1 | 0 | |
| **33e** | Sun Protection | | | 1 | 0 | |
| **33f** | Other(s): | | | | | |

Thank you for taking this survey!
